# Pulmonary actinomycosis during the first decade of 21st century: cases of 94 patients

**DOI:** 10.1186/1471-2334-13-216

**Published:** 2013-05-14

**Authors:** So Ri Kim, Lae Young Jung, In-Jae Oh, Young-Chul Kim, Kyeong-Cheol Shin, Min Ki Lee, Sei-Hoon Yang, Hee Sun Park, Mi-Kyung Kim, Jin Young Kwak, Soo-Jung Um, Seung Won Ra, Woo Jin Kim, Seungsoo Kim, Eu-Gene Choi, Yong Chul Lee

**Affiliations:** 1 Department of Internal Medicine and Research Center for Pulmonary Disorders, Chonbuk National University Medical School, San 2-20, Geumam-dong, Deokjin-gu, Jeonju, Jeonbuk, 561-180, Korea; 2 Department of Internal Medicine and Research Center for Pulmonary Disorders, Research Institute of Clinical Medicine of Chonbuk National University-Biomedical Research Institute of Chonbuk National University Hospital, San 2-20, Geumam-dong, Deokjin-gu, Jeonju, Jeonbuk, 561-180, Korea; 3 Department of Internal Medicine, Chonnam National University Medical School, Gwangju, Korea; 4 Department of Internal Medicine, Yeungnam University College of Medicine and Regional Center for Respiratory Disease, Yeungnam University Medical Center, Daegu, Korea; 5 Department of Internal Medicine, Pusan National University School of Medicine, Busan, Korea; 6 Department of Internal Medicine, Division of Pulmonary and Critical Care Medicine, School of Medicine, Wonkwang University, Iksan, Korea; 7 Department of Internal Medicine, Chungnam National University Medical School, Daejeon, Korea; 8 Department of Internal Medicine, Chungbuk National University College of Medicine, Cheongju, Korea; 9 Department of Internal Medicine, Presbyterian Medical Center, Jeonju, Korea; 10 Department of Internal Medicine, Division of Respiratory Medicine, Dong-A University, College of Medicine, Busan, Korea; 11 Department of Internal Medicine, Ulsan University Hospital, University of Ulsan College of Medicine, Ulsan, Korea; 12 Department of Internal Medicine, Kangwon National Univsersity School of Medicine, Chuncheon, Korea; 13 Department of Internal Medicine, College of Medicine, Division of Pulmonology, The Catholic University of Korea, Seoul, Korea; 14 Department of Internal Medicine, Konyang University Hospital, Daejeon, Korea

**Keywords:** Actinomycosis, Lung, Hemoptysis, Diagnosis, Treatment

## Abstract

**Background:**

Pulmonary actinomycosis is a chronic pulmonary infection caused by *Actinomyces*. Both improving oral hygiene and early application of antibiotics to the case of suspicious pulmonary infections result in changes in incidences and presentations of pulmonary actinomycosis. However, there are little reports dealt with the recent clinical characteristics of pulmonary actinomycosis. This study aimed to review the characteristics of pulmonary actinomycosis occurred during the first decade of 21st century.

**Methods:**

This retrospective study was performed on 94 subjects with pulmonary actinomycosis diagnosed pathologically from January 2000 to December 2010 in 13 hospitals in Korea.

**Results:**

The data of the study showed that pulmonary actinomycosis occurs frequently in middle to old-aged males (mean age; 57.7 years old) and that the most common symptoms are cough, hemoptysis, and sputum production. Various radiologic features such as the consolidation with central low attenuation (74.5%) and no regional predominance were also observed. Most of patients recovered completely with medical and/or surgical treatment, reaching approximately 98% cure rate.

**Conclusions:**

The results demonstrate that pulmonary actinomycosis is one of the cautious pulmonary diseases. More importantly, in cases of persistent hemoptysis or for differential diagnosis from lung malignancy, our data have revealed that surgical resection appears to be a useful intervention and that radiologic diagnosis may not provide decisive information. These findings indicate that it is important for the clinicians to include pulmonary actinomycosis as one of differential diagnoses for refractory pulmonary abnormal lesions to the current usual management.

## Background

Actinomycosis is a rare, chronic, and slowly progressive bacterial infection that induces suppurative and granulomatous inflammation characterized by swelling with suppuration, sinus tract formation, and purulent discharge containing yellowish sulfur granules [1]. Among six pathogenic species of *Actinomyces* spp., *Actinomyces israelii* is the most common human pathogen. The organism is filamentous, branching, Gram-positive, pleomorphic nonspore-forming, nonacid-fast anaerobic or facultative bacillus [2]. Although it is usually involved in oral and cervicofacial infection, other sites in the body such as abdominopelvic, thoracic, central nervous, and musculoskeletal systems can be infected [1].

The pulmonary actinomycosis represents approximately 15% of the total burden of disease [3]. The pulmonary actinomycosis is caused by aspiration of oropharyngeal or gastrointestinal secretions into the respiratory tract [4] and commonly presents as pulmonary infiltrate or a mass [5]. The most common symptoms are chest pain, productive cough, and dyspnea [6]. These non-specific clinical and radiologic presentations make pulmonary actinomycosis difficult to be diagnosed and often lead to misinterpretation as malignancy rather than an infective process [2,3]. In fact, even among experienced physicians, delayed diagnosis or misdiagnosis as tuberculosis, lung abscess or lung cancer is common [3]. In addition, both improvement of oral hygiene and early application of antibiotics to the case of suspicious pulmonary infections result in changes in an incidence and presentations of pulmonary actinomycosis, i.e., the incidence is decreased and the clinical features are less aggressive compared with pre-antibiotic era [3]. Microbiological and pathological examinations are indispensable for a correct diagnosis. However, to detect actual bacteria, the specimen should be collected prior to antimicrobial therapy and carefully transported to the laboratory in anaerobic media, avoiding contaminations with other external bacteria [2]. Therefore, the histopathologic finding of yellowish sulfur granules is often necessary for differential diagnosis of actinomycosis [7,8]. As for the treatment, pulmonary actinomycosis has been known to well respond to penicillin and cure without further therapeutic modality such as surgery [3,9].

Several studies have reported the data on pulmonary actinomycosis as a rare pathologic condition or reviewed clinical experiences, focusing on diagnosis, treatment, and outcome [5,7,8,10-25]. However, these reports hardly cover up the recent changes in pulmonary actinomycosis, specifically since 2000. In addition, the majority of studies have dealt with the data from relatively small number of patients (Table 1).

**Table 1:** Previous reports of pulmonary actinomycosis in English literatures since 1980

**Author**	**Nation**	**Year**	**No. of patients**	**Main issue**
Newsom et al. [12]	U.S.A.	1982	7	Diagnosis, appropriate drug therapy, surgical intervention
Jensen et al. [13]	Denmark	1989	9	Diagnosis, treatment
Kinnear et al. [14]	U.K.	1990	19	Clinical presentation, diagnosis, treatment
Kwong et al. [15]	Canada	1992	8	CT finding
Hsieh et al. [16]	Taiwan	1993	17	Clinical diagnosis, management
Rizzi et al. [8]	Italy	1996	13	Surgical consideration
Tastepe et al. [7]	Turkey	1998	7	Operation for diagnosis, treatment
Cheon et al. [17]	Korea	1998	22	CT finding
Dujneungkunakorn et al. [18]	Thailand	1999	16	Diagnosis, treatment
Yew et al. [19]	Hong Kong	1999	8	Treatment with imipenem
Baik et al. [20]	Korea	1999	25	Diagnosis, treatment
Endo et al. [25]	Japan	2002	13	Surgical intervention
Mato et al. [21]	Japan	2003	11	Clinical, radiological, pathological finding
Lu et al. [5]	Taiwan	2003	14	Surgical resection
Choi et al. [22]	Korea	2005	28	Duration of antibiotics
Kolditz et al. [23]	Germany	2009	49	Medical management
Song et al. [24]	Korea	2010	40	Treatment

In this study, we present a case series of 94 patients with pulmonary actinomycosis diagnosed pathologically from 2000 to 2010. We reviewed these cases, focusing on diagnosis, clinical features, radiological findings, and the need of therapeutic surgical intervention.

## Methods

### Patients

Ninety-four subjects with pulmonary actinomycosis diagnosed pathologically from January 2000 to December 2010 in 13 hospitals in Korea (Chonbuk National University Hospital, Chonnam National University Hospital, Yeungnam University Medical Center, Pusan National University Hospitial, Wonkwang University Hospital, Chungnam National University Hospital, Chungbuk National University Hospital, Presbyterian Medical Center, Dong-A University Hospital, Ulsan University Hospital, Kangwon National Univsersity Hospital, Daejeon St. Mary’s Hospital, and Konyang University Hospital) were analyzed retrospectively.

All patients were diagnosed as pulmonary actinomycosis based on the histopathologic identification of organisms from the tissues obtained by percutaneous transthoracic needle biopsy/aspiration (PTNB/A), resection by video-assisted thoracic surgery, wedge resection of lung, lobectomy, or bronchoscopic biopsy.

Patients’ medical records and chest radiographic images, including computed tomography (CT) scans were reviewed retrospectively. We analyzed the clinical information including baseline characteristics, clinical manifestations, diagnostic methods, therapeutic methods, and follow-up data. All patients were human immunodeficiency virus (HIV)-negative and did not have any diseases that compromise host’s immunity. The study was approved by Institutional Review Board of Choubuk National University Hospital (IRB file No. 2012-04-027-001).

### Image studies

All radiologic images were reviewed by experienced radiologists. The number, size, and distribution of lesions, presence of cavitation, calcification within nodule, and characteristics of margin were recorded. In addition, the radiologic initial diagnoses were collected.

### Histochemical staining

All patients were confirmed to have branching filamentous organisms, *Actinomyces* by Gomori’s methenamine silver staining and/or typical sulfur granules on hematoxylin-eosin staining.

### Classification of therapeutic responses

Therapeutic responses were classified into cure/complete recovery and treatment failure. ‘Cure’ was defined as a radiologic complete resolution, fibrotic inactive radiologic lesions or post-operation scarring and no related clinical symptoms. ‘Failure’ was defined as persistence or progression demonstrated by clinical or radiographic evidence of disease, despite medical and/or surgical therapy.

### Statistical analysis

All results were expressed as the mean and range or the number (percent) of patients.

## Results

### Patient characteristics and clinical manifestations

**Figure 1:**
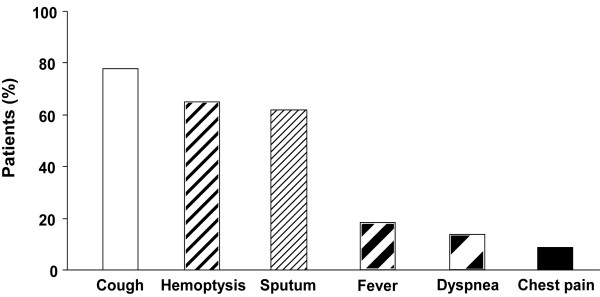
**Clinical manifestation of pulmonary actinomycosis.** The most common presenting symptoms were cough (77.7%), hemoptysis (64.9%), and sputum (61.7%).

Patient characteristics are summarized in Table 2. The median age was 57.7 years (31–83 years); 66 males (70.2%) and 28 females (29.8%) were included. Forty four patients were never-smokers, and 45 patients had underlying pulmonary conditions such as history of infections by mycobacteria (n = 21), chronic obstructive pulmonary disease (n = 10), bronchiectasis (n = 18), and aspergillosis (n = 8). As for the inflammatory parameters, 26 patients showed the elevated levels of leukocytes (> 10.8 × 10^3^/ml), and 25 patients had the raised levels of CRP (> 5 mg/L). In addition, 43 patients showed the elongated ESR > 20 mm/hr. The most common presenting symptoms were cough (77.7%), hemoptysis (64.9%), and sputum production (61.7%) as presented in Figure 1.

**Table 2:** Baseline characteristics of 94 patients with pulmonary actinomycosis

	**Number (%) or mean (range)**
Age (years)	57.7 (31–83)
Gender, male	66 (70.2%)
Never-smoker	44 (46.8%)
**Co-morbidity disease(pulmonary)**	
Pulmonary Tuberculosis or NTM	21 (22.3%)
COPD	10 (10.6%)
Bronchiectasis	18 (19.1%)
Aspergillosis	8 (8.5%)
Lung cancer	4 (4.3%)
**Co-morbidity disease(Non-pulmonary)**	
Alcohol abuse	16 (17.0%)
Diabetes	18 (19.1%)
Hypertension	18 (19.1%)
Ischemic heart disease	2 (2.1%)
**Laboratory finding & Pulmonary function test**	
WBC count (/μL, × 10^3^)	10.10 ± 3.95
ESR (mm/hr)	55.9 ± 39.1
CRP (mg/L)	11.8 ± 27.6
FEV1 (L)	2.22 ± 0.79
FEV1 (%)	80.6 ± 26.4

### Image findings

**Figure 2:**
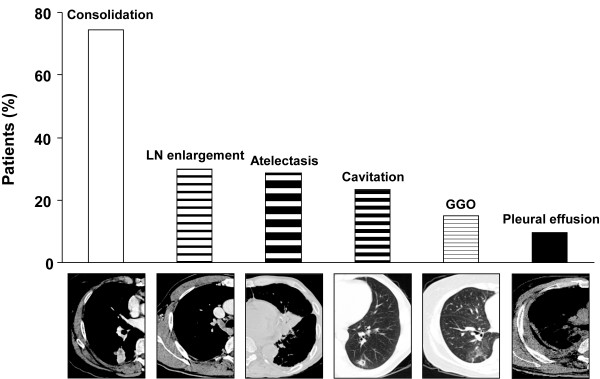
**CT findings of pulmonary actinomycosis.** The most common chest CT finding was consolidation (74.5%), followed by mediastinal or hilar lymph node enlargement (29.8%), atelectasis (28.7%), cavitation (23.4%), and ground-glass opacity (14.9%). GGO: ground glass opacity, LN: lymph node.

Simple chest X-ray and CT were available for all patients. The most common chest CT finding (Figure 2) was consolidation (74.5%), followed by mediastinal or hilar lymph node enlargement (29.8%), atelectasis (28.7%), cavitation (23.4%), and ground-glass opacity (14.9%). Pleural effusion was also presented in 8 patients (9.6%).

### Initial diagnoses

As expected, the majority of patients were misdiagnosed initially (Table 3). On the basis of clinical and radiological findings, the most common initial diagnosis was lung cancer (35.1%), followed by pneumonia (19.1%), mycobacterium infection (17.0%), aspergillosis (8.5%), and lung abscess (5.3%). Only 6 cases (6.4%) were diagnosed as pulmonary actinomycosis.

**Table 3:** Initial radiologic diagnoses of 94 cases

**Initial diagnosis**	**Number (%)**
Lung cancer	33 (35.1%)
Pneumonia	18 (19.1%)
Pulmonary Tuberculosis or NTM	16 (17.0%)
Aspergillosis	8 (8.5%)
Actinomycosis	6 (6.4%)
Lung abscess	5 (5.3%)
Empyema	3 (3.2%)
Broncholithiasis	2 (2.1%)
Granuloma	2 (2.1%)
Fibrothorax	1 (1.0%)

### Diagnostic modalities

For pathologic confirmation, various invasive and semi-invasive methods were used to obtain the tissues of pulmonary lesions. Forty seven patients (50.0%) were underwent surgical biopsy, and PTNB/A and bronchoscopic biopsy were performed in 23 patients and 24 patients, respectively.

### Treatment and clinical outcomes

Empirical antibiotics were used for the patients before the diagnosis was confirmed as pulmonary actinomycosis. Several antibiotics were selected to treat pulmonary actinomycosis such as penicillin G, cephalosporin, ampicillin, and amoxillin. Among them, cephalosporins used to treat our patients are as followed; ceftriaxone, cefpiramide, ceftizoxime, cefmetazole, cefminox, cefetamet, cefirad, cefotaxime, cefotiam, and cefoxitin. The antibiotic treatment was initiated intravenously after diagnosis of actinomycosis and followed by oral antibiotics. The mean duration of treatment with intravenous antibiotics and oral antibiotics are 14.7 days (range; 1–56 days) and 153.2 days (range; 5–672 days), respectively. Table 4 listed the intravenous antibiotics used to treat our patients. Interestingly, the frequently selected intravenous antibiotics were different between two groups. In surgical group, cephalosporin was used as the antibiotic treatment in a large number of patients (38.8%, 19 patients from 49 patients), on the other hand, penicillin G was most frequently chosen for the treatment of the patients without surgery (44.4%, 20 patients from 45 patients) (Table 5).

**Table 4:** List of intravenous antibiotics used for treatment

**Antibiotics**	**Number (%)**
Penicillin G	30 (34.1%)
Cephalosporin	24 (27.3%)
Ampicillin/Sulbactam	7 (7.9%)
Amoxicillin	5 (5.7%)
Piperacillin/Tazobactam	4 (4.5%)
Piperacillin/Sulbactam	1 (1.1%)
Cabapenem	1 (1.1%)
Erythromycin	1 (1.1%)
Quinolone	1 (1.1%)
Aminoglycoside	1 (1.1%)
Others	13 (14.8%)

**Table 5:** Comparison of intravenous antibiotics used between patients with surgery and without surgery

**Patients with surgery (n = 49)**		**Patients without surgery (n = 45)**	
Cephalosporin	19 patients	Penicillin G	20 patients
Penicillin G	10 patients	Cephalosporin	5 patients
Ampicillin/sulbactam	3 patients	Ampicillin/sulbactam	4 patients
Amoxicillin	3 patients	Amoxicillin	2 patients
Piperacillin/Tazobactam	3 patients	Piperacillin/Tazobactam	1 patient
Levofloxacin	1 patient	Aminoglycoside	1 patient
Erythromycin	1 patient		
Cabapenem	1 patient		
Piperacillin/Sulbactam	1 patient		
No record	0 patients	No record	2 patients
No antibiotic treatment	3 patients	No antibiotic treatment	1 patient*
No intravenous antibiotic treatment	4 patients	No intravenous antibiotic treatment	9 patients

* The patient presented as an asymptomatic pulmonary nodule was spontaneously cured during the diagnostic process.

As for surgical resection, 49 patients (52.1%) underwent the surgery for the control of persistent hemoptysis (n = 22), no radiologic response despite medical treatment (n = 4), the treatment of combined disorders such as aspergilloma (n = 3), empyema (n = 2), or fibrothorax (n = 1), and differential diagnosis from lung malignancy (n = 17).

Ninety-two patients were completely recovered by the medical and/or surgical treatment, while only two patients died due to complications. The structural serious complication of pulmonary actinomycosis was not developed except only one patient who was expired due to broncho-pleural fistula.

## Discussion

In the present study, we analyzed the recent ten-year data on pulmonary actinomycosis from 13 medical centers scattered in Korea and found that common clinical manifestations are cough, hemoptysis, and sputum; that consolidation is the predominant radiologic feature on CT scan; that the majority cases are initially diagnosed as lung cancer or pneumonia; and that pulmonary actinomycosis is well responded to antibiotic therapy when they are not combined with hemoptysis or severe complicated sequelae such as broncho-pleural fistula. In fact, surgical treatment was helpful for the persistent hemoptysis despite the antibiotic therapy.

Pulmonary actinomycosis can develop in both sexes and at all ages, although previous reports have shown that it is more prevalent in males aged 30 to 50 years [1,14]. In our current study, the patients were slightly older than those in the previous studies; the male patients were predominant (70.2%) and mean age was 57.7 years old, ranging 31 to 83 years old. Many cases had the structural lung diseases, including the history of mycobacterium infection and bronchiectasis. In addition, approximately one fifth of patients were alcohol abusers. These findings support the general contention that pulmonary actinomycosis is mainly caused by aspiration of oral substances.

Interestingly, pulmonary actinomycosis seems to be more often reported in Korea than in other regions. To date, the accurate prevalence or incidence rate of pulmonary actinomycosis is little known globally as well as in Korea. This study could not provide this information, either. However, in Korea, with the technical improvements of CT resolution and the increasing use of CT scan for health screening as well as for specific diagnosis, the detection rate of pulmonary actinomycosis seems to become higher than before. In addition, the cost of CT scan is relatively cheaper in Korea than in other countries, thank to Korean National Health Insurance. The technical improvements and low cost seem to contribute to the recent inclination of the report on pulmonary actinomycosis in Korea.

Clinical manifestations of pulmonary actinomycosis are variable although cough and sputum are the most common symptoms [14,16]. Marked weight loss, malaise, and high fever may be presented in pulmonary actinomycosis, especially in systemic disseminated cases, however, the early application of antibiotics reduces these serious presentations [10]. In fact, these presentations can be changed with time in line with the decrease in the disease prevalence [26]. The most common presentation is the presence of a shadow on a chest radiograph. Interestingly, our data revealed that hemoptysis is one of common clinical features in pulmonary actinomycosis. Although there are considerable debates on this issue, a previous report has also described that the commonest clinical manifestation of pulmonary actinomycosis is hemoptysis in Korea [20]. Moreover, our data revealed that the occurrence rate of hemoptysis in patients with pulmonary comorbidities (71.3%) was higher than the rate in patients without pulmonary comorbidities (56.3%). Based on these observations, it is possible that the high incidence of hemoptysis in our patients with pulmonary actinomycosis is due to the underlying structural diseases such as pulmonary tuberculosis, bronchiectasis, and aspergilloma, which can frequently develop hemoptysis and are relatively prevalent in Korea. In fact, in our some cases, persistent hemoptysis was occurred during antibiotic treatment, and to control bleeding, these patients underwent bronchial artery embolization or thoracotomy. These findings suggest that actinomycosis should be suspected in patients with hemoptysis of unknown cause.

The radiological findings of pulmonary actinomycosis can resemble a broad spectrum of lung pathologies from benign infection to metastatic tumor [27-29]. For many physicians, the main problem is distinguishing the disease from a neoplasm [30]. Actually, on simple chest radiographs, consolidation or mass-like lesion is common [27]. On chest CT scan, pulmonary actinomycosis can be presented as a patchy air-space consolidation, nodular appearance with central low attenuation, pleural thickening, empyema or lymphadenopathy. The disease usually shows peripheral and lower lobe predominance, probably reflecting the role of aspiration in its pathogenesis [15]. In our current study, mass-like lesions or consolidations were the most common CT findings; the majority of consolidations showed the central low attenuation. However, there was no significant predominance in specific sites. Other radiologic findings include cavitation, lymphadenopathy, and ground glass opacity.

Diagnosis of pulmonary actinomycosis involves imaging modalities as a relatively easy and convenient approach. However, invasive procedures such as bronchoscopic, thansthoracic or even surgical biopsies are required for histological and microbiological examination to confirm the diagnosis. In fact, the majority of patients in our current study were initially misdiagnosed as lung cancer, mycobacterium infection, or pneumonia based on the radiological imaging findings. We recruited the patients with pulmonary actinomycosis diagnosed pathologically through surgery, PTNB/A, and bronchoscopic biopsy. Among them, a half of patients were diagnosed correctly by semi-invasive procedures such as PTNB/A (25.5%) or bronchoscopic biopsy (24.5%). Estimating the risk/benefit ratio, these semi-invasive procedures are clinically useful and safe for the diagnosis of pulmonary actinomycosis, although 17 patients underwent surgical resection for the final diagnosis.

Treatment of pulmonary actinomycosis with antibiotics is highly effective, and early diagnosis is more likely to lead to cure [20]. Administration of high-dose intravenous penicillin for a long duration is the primary treatment. Although the use of penicillin has to be modified depending on the individual conditions, a recommended dose is 18–24 million units of penicillin per day for 2–6 weeks, followed by oral therapy with penicillin V (or amoxicillin) for 6–12 months [10]. For the patients allergic to penicillin, erythromycin, tetracycline, or clindamycin is a possible alternative. In our cases, 98% of the patients were completely cured by administration of these antibiotics. In addition, we found that the percent of patients treated with cephalosporins was greater than that of other previous clinical reports. Intriguing point is that the majority of patients treated with cephalosporins underwent surgery, while penicillin G was most frequently chosen to treat the patients without surgery. These findings suggest that the classic antibiotic regimen, penicillin G is preferred for treating patients without surgery and that the effectiveness of cephaloscporins for pulmonary actinomycosis is comparable to penicillin G for the patients who underwent surgery. Meanwhile, we also found that approximately one fifth of patients treated medically were treated with oral antibiotics only, following the favorable clinical outcome. Although they took the oral antibiotics for a longer period of time than those treated with both intravenous and oral antibiotics, the dose prescribed did not differ from the usual level. These findings suggest that some selected patients with pulmonary actinomycosis, especially exhibiting mild disease severity and no complications, can be treated with oral antibiotics only at the outpatient department.

However, during the antibiotic treatment, our current study revealed that 22 patients with hemoptysis did not respond to the antibiotics, therefore, they underwent surgery for the bleeding control. A recent study has reported that patients who underwent surgical intervention can have more favorable clinical outcome compared to the patients treated with antibiotics [24]. In addition, our two patients were expired due to massive hemoptysis or respiratory distress combined with broncho-pleural fistula, which are known as serious complications of pulmonary actinomycosis. Based on these experiences, we can carefully recommend that surgical intervention should be considered early and actively as the therapeutic modality for complicated pulmonary actinomycosis, despite the excellent outcomes of the treatment with antibiotics.

In this paper, we have desired to describe useful clinical and radiological information on pulmonary actinomycosis based on relatively large sample sized medical data, however, there are some limitations including retrospective study design, inclusion of patients diagnosed by pathologic examination only, the shortage of data on microbial culture, and no follow up or recurrence data after the treatment. Therefore, we are waiting for a prospective large-scaled study for establishing of optimal therapeutic modalities and appropriate diagnostic methods for pulmonary actinomycosis and that this study will be a cornerstone for the future research.

## Conclusion

This present study reviewed the characteristics of pulmonary actinomycosis occurred during the first decade of 21st century. Pulmonary actinomycosis frequently develops in middle to old aged males, especially having the habit of alcohol abuse and structural lung diseases. While the clinical presentations are milder than those in the previous reports, hemoptysis is one of the cautious symptoms of pulmonary actinomycosis. The most common radiologic feature is non-specific consolidation resembling to lung malignancy or pneumonia. The semi-invasive procedure such as PTNB/A is very useful for its diagnosis. Although the clinical outcome for the antibiotic therapy is excellent, in cases of persistent hemoptysis or for differential diagnosis from lung malignancy, the surgical resection appears to be a useful intervention. Above all things, the suspicion of pulmonary actinomycosis by the physicians is most important for the appropriate management of the disease. Therefore, it is worthy to recommend for clinicians to consider pulmonary actinomycosis as one of differential diagnoses for refractory pulmonary abnormal lesions to current usual management.

## Abbreviations

CT: Computed tomography; GGO: Ground glass opacity; HIV: Human immunodeficiency virus; LN: Lymph node; PTNB/A: Percutaneous transthoracic needle biopsy/aspiration.

## Competing interest

The authors declare that they have no competing interests.

## Authors’ contribution

Kim SR designed research, interpreted data, and wrote the manuscript; Jung LY designed research, analyzed data, and drafted the manuscript; Oh IJ, Kim YC, Shin KC, Lee MK, Yang SH, Park HS, Kim MK, Kwak JY, Um SJ, Ra SW, Kim WJ, Kim SS, Choi EG gathered and analyzed data; Lee YC designed research, interpreted data, and edited the manuscript. All authors read and approved the final manuscript.

## Pre-publication history

The pre-publication history for this paper can be accessed here:

http://www.biomedcentral.com/1471-2334/13/216/prepub
